# Safety of Naloxone in Opioid-Naïve Methadone Intoxicated Patients; a Case Series Study 

**Published:** 2020-03-02

**Authors:** Seyed Hamid Reza Shakeri, Hossein Hassanian-Moghaddam, Nasim Zamani

**Affiliations:** 1 Department of Emergency Medicine, Mazandaran University of Medical Sciences, Sari, Iran.; 2 Social Determinants of Health Research Center, Shahid Beheshti University of Medical Sciences, Tehran, Iran.; 3 Department of Clinical Toxicology, Loghman Hakim Hospital, School of Medicine, Shahid Beheshti University of Medical Sciences, Tehran, Iran.

**Keywords:** Naloxone, Substance Withdrawal Syndrome, Analgesics, Opioid, Drug-Related Side Effects and Adverse Reactions

## Abstract

**Introduction:**

Studies have shown that naloxone can cause behavioral changes in naïve normal volunteers. This study aimed to investigate the possible complications of naloxone in methadone-overdosed opioid-naïve patients.

**Methods:**

In this pilot study, a total number of 20 opioid-naïve methadone-poisoned patients underwent naloxone challenge test to receive naltrexone. 0.2, 0.6, and 1.2 mg doses of naloxone were administered on minutes 0, 5, and 15-20. The patients were followed for 30 minutes after administration of naloxone and monitored for any upsetting signs and symptoms. Patients with clinical opiate withdrawal scale (COWS) lower than 5 were considered not addicted and the severity of patients’ symptoms was calculated using subjective opiate withdrawal syndrome (SOWS).

**Results:**

20 patients with mean age of 25.5±8.09 years were evaluated (70% female). Median ingested dose of methadone was 25 mg [IQR; 10 to 50 mg] and mean time interval between ingestion of methadone and naloxone challenge test was 7.1±4.9 hours. Fourteen patients reported some discomfort after administration of a mean dose of 1.7±0.5 mg of naloxone lasting for a maximum of four hours. The most common patients’ complaints were headache (45%) followed by nausea (20%), agitation (20%), abdominal pain (20%), and flushing (20%). Two (10%) mentioned severe panic attack and sensation of near-coming death. SOWS significantly correlated with female gender (p = 0.004) and time elapsed post methadone ingestion (p = 0.001).

**Conclusion:**

It seems that naloxone is not a completely safe medication even in opioid-naïve patients, and administrating adjusted doses of naloxone even in opioid-naïve methadone intoxicated patients may be logical.

## Introduction

Naloxone is a safe and effective rescue medication for opioid overdose, a leading cause of death, worldwide. It blocks the opioid receptors for 30-90 minutes to reverse the respiratory depression that would otherwise lead to apnea and death.

Serious side effects of naloxone are very rare. The most common side effect is opioid withdrawal in dependent patients, risk of which increases with larger doses of naloxone as well as the strength of drug dependency (1). Although generally regarded to be safe, naloxone may precipitate rare side effects including body aches, fever, sweating, sneezing, yawning, nausea/vomiting, lacrimation, rhinorrhea, cramping, insomnia, chills, hot flashes, tachycardia, restlessness/irritability, tremulousness, seizure, hypo or hypertension/arrhythmias in patients with cardiac disorders, dizziness, headache, tremor, and pulmonary edema (2).

Although much more common in dependent patients, previous studies have shown that in naïve normal volunteers, high-dose naloxone (2-4 mg/stat) can cause "rush" or "buzz", tingling/numbness of the extremities, dizziness, reluctance to move or initiate activities, abdominal pain, impaired cognitive performance, and increased tension-anxiety, anger-hostility, depression-dejection, and confusion-bewilderment subscales, which could last for hours (3). These symptoms were then attributed to blockage of the endogenous opioids (3). 

Naloxone may be used to perform naloxone challenge test before administration of naltrexone, as a long term antagonist of the opioid, in opioid-naïve patients (4). Since naltrexone may cause life threatening conditions in opioid-dependent patients, it is essential to confirm naivety before its administration (5). While performing this practice, we noticed that some opioid-naïve patients under the effect of long-acting opioids experienced severe discomfort. This study aimed to investigate the possible complications of naloxone in methadone-overdosed opioid-naïve patients. 

## Methods

***Study design and setting***

In this case series study, opioid-naïve methadone-poisoned patients who were referred to emergency department of Loghman Hakim Hospital, Tehran, Iran, and underwent naloxone challenge test to receive naltrexone during a 3-month period between March and June 2019 were evaluated. The experiment was conducted with the informed consent of the human subject. Ethical approval for the study was given by Shahid Beheshti University of Medical Science Ethics Committee (Ethic code: 1391-1-113-9658).

***Participants***

Patients who claimed not to be opioid dependent and had accidentally or intentionally (suicidal or recreational) ingested methadone (based on the obtained history), were included. The participants were those who needed opioid-antagonist administration for long times due to persistent respiratory acidosis (pH<7.2 and PCO2 >52) (5). All had signs and symptoms of acute opioid toxicity and urine test results returned to be positive only for methadone. Those with active liver disease and increased level of transaminases (twice the normal range), acute poisoning with complications developed before presentation (aspiration pneumonia, brain trauma, and acute lung injury), and those discharged against medical advice were excluded. 

***Procedure***

In our center, the routine protocol to manage these patients is administration of naltrexone instead of naloxone to avoid long naloxone infusions and hospital stay. However, before administration of naltrexone it is necessary to make sure the patient is not opioid-dependent as he/she may not give the correct history on being naïve.

We administered 0.2, 0.6, and 1.2 mg doses of naloxone in minutes 0, 5, and 15-20 (6, 7). The patient was followed for 30 minutes after administration of naloxone doses and monitored for signs and symptoms including nausea and vomiting, agitation, flushing, abdominal pain and cramp, anxiety and restlessness, muscle and bone pain, and panic attack. 

***Data gathering***

Possible side effects as well as the patients’ characteristics (age and gender), amount of the ingested methadone, duration of the symptoms lasting after administration of naloxone, total naloxone dose administered, and treatments given were recorded by a toxicology fellow using a predesigned checklist. 

To confirm that the patient was not addicted, clinical opiate withdrawal scale (COWS) was determined (8) and scores lower than 5 were considered not addicted. To determine the severity of patients’ symptoms, subjective opiate withdrawal syndrome (SOWS) was calculated (9). 

***Statistical analysis***

The data was analyzed using statistical package for social sciences (SPSS) version 20 using Kolmogorov-Smirnov test, t-test, Mann-Whitney U test, and Spearman test. P < 0.05 was considered significant. Data are presented as mean ± standard deviation, median and inter quartile range (IQR), or frequency (%).

## Results

***Baseline characteristics of studied patients***

20 patients with the mean age of 25.5±8.09 (range; 17 to 46) years were evaluated (70% female). Thirteen (75%) had overdosed on methadone syrup and 7 (35%) had consumed methadone tablets. Median ingested dose of methadone was 25 mg [IQR; 10 to 50 mg] and mean time between ingestion of methadone and naloxone challenge test was 7.1±4.9 (range; 2 to 24) hours. 18 (90%) patients had referred with loss of consciousness and 9 (45%) had respiratory acidosis on presentation (pH < 7.3 and PCO2 > 54mmHg). Mean pH and PCO2 on admission were 7.2 ± 0.05 (range; 7.12 to 7.38) and 51.4 ± 3.5 (range; 46 to 58) mmHg, respectively.

***Side effects***

**Figure 1:**
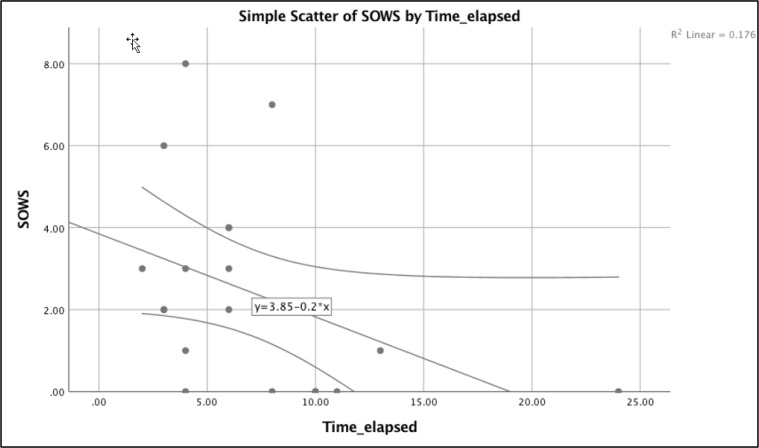
Scatter plot showing the relationship between time post methadone ingestion and severity of subjective opiate withdrawal syndrome (SOWS).

14 (70%) patients reported some discomfort after administration of a mean dose of 1.7 ± 0.5 mg of naloxone (0.2 to 2 mg). Their symptoms lasted for a maximum of four hours posttest. The most common symptom the patients complained about was headache (45%) followed by nausea (20%), agitation (20%), abdominal pain (20%), and flushing (20%). 2 (10%) cases mentioned severe panic attack and sensation of near-coming death. Mean COWS and SOWS scores were 2.4±1.9 and 2.4±2.4, respectively. SOWS significantly correlated with female gender (t test; p = 0.004) and time elapsed post methadone ingestion (spearman rho test; p=0.001; Figure 1). 

## Discussion

Naloxone is a derivative of natural plant alkaloid and is similar to oxymorphone.  Naloxone, however, is a competitive antagonist of the opiate receptors and has particularly high affinity for the μ opiate receptor and can displace morphine and other full agonists, thereby reversing their effects (10). 

In 2015 the American Association of Poison Control Centers reported no fatalities due to naloxone other than buprenorphine/naloxone combinations (11). Although post naloxone administration complications had been previously reported in opioid-naïve normal volunteers (3), these symptoms were mainly behavioral and had been attributed to the effect of the endo-morphines.

This is the first study reporting complications after administration of naloxone in methadone-overdosed opioid-naïve patients. The low COWS score shows that our patients were not really opioid dependent. However, they showed a series of inadvertent reactions after naloxone administration. This may be due to mu receptors developing tolerance during the time elapsed between methadone ingestion and naloxone administration. In fact, it seems that the receptors have justified themselves to the high methadone concentration, while administration of naloxone reverses this transient tolerance and causes patient discomfort. This may show that despite what was previously alluded naloxone is not a very safe medication even in opioid-naïve patients. Therefore, administrating adjusted doses of naloxone even in naïve patients seems logical.

We had a small sample size because we ran a pilot on only 20 patients. Future studies on larger sample sizes are warranted to better elucidate the effect of naloxone on opioid-naïve patients. 

## Conclusion:

It seems that naloxone is not a completely safe medication even in opioid-naïve patients, and administrating adjusted doses of naloxone even in opioid-naïve methadone intoxicated patients may be logical.

## References

[1] Naloxone: Frequently Asked Question.

[2] Naloxone Side Effects.

[3] Cohen M, Cohen R, Pickar D, Weingartner H, Murphy D, Bunney JR W (1981). Behavioural effects after high dose naloxone administration to normal volunteers. The Lancet.

[4] Aghabiklooei A, Hassanian-Moghaddam H, Zamani N, Shadnia S, Mashayekhian M, Rahimi M (2013). Effectiveness of naltrexone in the prevention of delayed respiratory arrest in opioid-naive methadone-intoxicated patients. BioMed Research International.

[5] Hamdi H, Hassanian-Moghaddam H, Hamdi A, Zahed NS (2016). Acid-base disturbances in acute poisoning and their association with survival. Journal of critical care.

[6] Khosravi N, Zamani N, Hassanian-Moghaddam H, Ostadi A, Rahimi M, Kabir A (2017). Comparison of Two Naloxone Regimens in Opioid-dependent Methadone-overdosed Patients: A Clinical Trial Study. Current clinical pharmacology.

[7] Ostadi A, Zamani N, Hassanian-Moghaddam H, Khosravi N, Shadnia S (2019). Comparison of 2 Naltrexone Regimens in the Maintenance Therapy of Acute Methadone Overdose in Opioid-Naïve Patients: A Randomized Controlled Trial. Crescent Journal of Medical and Biological Sciences.

[8] Clinical opiate withdrawal scale.

[9] Subjective opiate withdrawal scale.

[10] Naloxone.

[11] Mowry JB, Spyker DA, Brooks DE, Zimmerman A, Schauben JL (2016). 2015 Annual Report of the American Association of Poison Control Centers’ National Poison Data System (NPDS): 33rd Annual Report. Clinical Toxicology.

